# Effects of the response to the COVID-19 pandemic in chest trauma patients in China: a multicenter retrospective study

**DOI:** 10.1186/s13019-023-02463-3

**Published:** 2023-11-30

**Authors:** Zhengwei Wang, Mi Li

**Affiliations:** Department of Thoracic Surgery, The 904th Hospital of PLA Joint Logistic Support Force, Xing Yuan North Road 101, Wuxi, 214044 China

**Keywords:** COVID-19, Chest trauma, Outcome, Epidemiology

## Abstract

**Background:**

An international pandemic of severe coronavirus disease (COVID-19) has been caused by the novel coronavirus SARS-CoV-2. A large number of patients with chest trauma were infected with COVID-19. The COVID-19 pandemic had a significant impact on the management of chest trauma.

**Objective:**

The present observational study was conducted to evaluate the clinical characteristics and outcomes of chest trauma patients with or without COVID-19 infection.

**Methods:**

A multicenter observational study was performed at three Chinese hospitals between November 1, 2022, and January 31, 2023. All enrolled patients were diagnosed with chest trauma. We analyzed data from existing medical records, including all baseline data and prognostic follow-up data, such as 30-day mortality, hospital stays, hospitalization costs, and complications.

**Results:**

All 375 eligible patients completed the follow-up. There was no significant difference in baseline characteristics between chest trauma combined with COVID-19 (*p* > 0.05). Chest trauma combined with COVID-19 infection may lead to higher 30-day mortality (16.36% vs. 7.14%, *p* = 0.005), longer hospital stays (22.5 ± 5.9 vs. 12.8 ± 4.2, *p* < 0.001), larger hospitalization costs (*p* < 0.001), and increased hospitalization complications, such as pulmonary embolism (10.30% vs. 4.76%, *p* = 0.039), deep vein thrombosis (DVT, 33.33% vs. 18.57%, *p* = 0.001), the incidence of 7-day delirium (69.70% vs. 46.19%, *p* < 0.001), and respiratory failure (38.18% vs. 24.77%, *p* = 0.005).

**Conclusions:**

Compared to chest trauma alone, it may lead to higher mortality, larger hospitalization costs, and more complications. To better respond to the future of COVID-19 or other similar virus-borne disease pandemics, it is important to understand the clinical characteristics and complications, such as pulmonary embolism, DVT, and respiratory failure after COVID-19 infection. To guide the future treatment of chest trauma combined with COVID-19 infection or other infectious diseases.

## Introduction

Global changes in public health measures have been prompted by the COVID-19 pandemic caused by the novel coronavirus SARS-CoV-2, causing many patient deaths around the world [1]. Worldwide, COVID-19 has been challenging the healthcare system for nearly three years, affecting the management and treatment of intensive care unit patients. Compared with other countries, China took a different approach to outbreak response before December 2022, from the Wuhan lockdown in 2020 to the dynamic zero-COVID policy, employing precise prevention and control methods to stop the transmission of SARS-CoV-2 [2]. By December 2022, China is expected to adjust its prevention and control policies, and China's rapid outbreak of the epidemic has captured the attention of the world. The brief outbreak of SARS-CoV-2 has shaken China's medical industry and even led to the collapse of the medical system. A large number of patients with COVID-19 have entered hospitals. Then, a large number of patients with primary disease combined with COVID-19 infection appeared. However, there is little information about the risk of severe disease associated with COVID-19.

One of the most common causes of death in the world is trauma-related injuries, particularly severe multiple traumas combined with severe chest trauma or traumatic brain injuries [3, 4]. Severe traumatic stress can induce systemic inflammation and coagulation dysfunction, which leaves the patient vulnerable to infection. Patients with chest injuries often have lung injuries. SARS-CoV-2 pulmonary damage is characterized pathologically by diffuse alveolar damage and thrombosis. Furthermore, there is a possibility of nosocomial bacterial superinfections and ventilator-induced lung injury (VILI) [5]. As a result of contracting COVID-19, patients with external chest injuries will suffer more lung damage. There is strong evidence that traumatic brain injury is most prevalent in developing countries, where mortality rates are higher due to COVID-19 infection [6]. However, no researchers have reported chest trauma epidemiology and medical features in China during the SARS-CoV-2 pandemic.

Therefore, the objective of our research was to explore the impact of the SARS-CoV-2 pandemic on common risk factors for patients suffering from chest trauma.

## Materials and methods

### Study design and patients

The present study was a multicenter observational study performed at three Chinese hospitals (3A with a high-standard trauma center) between November 1, 2022, and January 31, 2023. Inclusion criteria were the diagnosis of chest trauma at admission, similar to previous studies on the SARS-CoV-2 pandemic’s impact on injury epidemiology [7]. The following patients were excluded: history of pulmonary surgery, history of pulmonary infection, lung tumor history, and those in remission from a lung tumor or if there was documented refusal. Patients were divided into two groups according to whether they were infected with COVID-19. Patients with chest trauma who presented to our trauma center as adults were collected for data collection. We analyzed all clinical baseline data, outcome data from existing medical records (sex, age, mechanism of injury, delirium, complications, survival situation, etc.), and management. Follow-up will be conducted online or by telephone (eight months) to clarify the patients’ survival situation. The research protocols were designed and conducted to measure the potential safety and efficacy in chest trauma patients after the SARS-CoV-2 pandemic. It was registered with the registration number CWXH-IPR-2022012 (date: 01/Nov 2022) with protocol approval from the Clinical Research Ethics Committees of 904th Hospital (Approval number: YXLL-2022-062).

### Outcome evaluation methods

In the present study, three Chinese hospitals recorded all patient outcomes and complication data. The delirium incidence was evaluated and continued twice a day during the first week [8]. We used CAM and CAM for the ICU (CAM-ICU) to detect 4 features: (1) acute appearance or a fluctuating course of changes in mental status, (2) inattention, (3) thinking disorganization, and (4) consciousness level alteration. Patients displaying features 1 and 2, with either 3 or 4, were diagnosed with developing delirium [8]. The diagnosis of pulmonary embolism was based on venous Doppler ultrasound and pulmonary artery CTA. The diagnosis of pulmonary embolism was made according to venous Doppler ultrasound. The diagnosis of other complications should be performed jointly by a specialist and a thoracic surgeon.

### Statistical analysis

The t-test was used for normally distributed data (mean ± SD), and the Mann‒Whitney U test was used for nonnormally distributed data (mean ± SD). The χ^2^ test or continuity correction χ^2^ test was used to compare the categorical data. We used R software (version 3.5.3) to analyze the survival analysis. Statistical significance was determined by *p* < 0.05. Mean differences and risk ratios were calculated with a 95% CI (confidence interval) on both sides. To analyze the data, IBM SPSS Statistics version 24 was used (version 20, IBM, Chicago, IL). Data oversight was performed by the 904th Hospital of the Joint Logistic Support Force of PLA.

## Results

### Baseline patient characteristics: overall population

A total of 392 chest trauma patients were screened, and 375 eligible patients were recruited and subjected to assessment between November 1, 2022, and January 31, 2023. COVID-19 testing for patients was performed using RT‒PCR. The mean age was 51.03 ± 8.69 years (range: 18–72 years). Females represented 42.1% of the population. A total of 165 patients had chest trauma combined with COVID-19, and the other 210 patients had chest trauma without COVID-19. We also found no significant difference between chest trauma combined with COVID-19 infection or not on baseline characteristics (Table 1).

**Table 1:** Comparison of baseline data

	COVID-19 (n = 165)	Non- COVID-19 (n = 210)	*p*
Age (Y, mean ± SD)	51.2 ± 8.8	50.9 ± 8.6	0.740
Gender, no. (%)			0.746
Male	93 (56.36%)	124 (59.05%)	
Female	72 (43.63%)	86 (40.95%)	
BMI (KG/cm^2^, mean ± SD)	22.1 ± 2.6	21.8 ± 2.3	0.237
Cause of disease, no. (%)			
Traffic Accident	90 (54.54%)	106(50.48%)	0.434
Falls	47 (28.48%)	67 (31.90%)	0.475
Violence	16 (9.70%)	27 (12.86%)	0.340
Others	12 (7.28%)	10 (4.76%)	0.304
Smoking History, no. (%)			0.680
Yes	68 (41.21%)	91 (43.33%)	
No	97 (58.89%)	119 (56.67%)	
Drinking History, no. (%)			0.404
Yes	84 (50.91%)	116 (55.24%)	
No	81 (49.04%)	94 (44.76%)	
Living environment, no. (%)			0.595
Town	98 (59.39%)	119 (56.67%)	
Countryside	67 (40.61%)	91 (43.33%)	
Past medical history, no. (%)			
Hypertension	52 (31.51%)	74 (35.23%)	0.449
Hyperlipidemia	60 (36.36%)	75 (35.71%)	0.897
Diabetes	47 (28.48%)	56 (26.67%)	0.695
Heart disease	17 (10.30%)	22 (10.48%)	0.957
Respiratory system disease	15 (9.09%)	17 (8.10%)	0.732
Flail chest	86 (52.12%)	105 (50.00%)	0.818
Hemothorax	78 (47.27%)	91 (43.33%)	0.447
Pneumothorax	92 (55.76%)	115 (54.76%)	0.847
ISS, mean ± SD	13.97 ± 1.65	14.15 ± 1.56	0.280
AIS	3.47 ± 0.61	3.42 ± 0.57	0.414
RTS, mean ± SD	7.82 ± 1.96	7.65 ± 1.82	0.386
Death probability TRISS, mean ± SD	0.89 ± 0.11	0.91 ± 0.14	0.133

*TRISS* trauma injury severityscore, *IQR* interquartile range, *AIS* abbreviated injury scale, *ISS* injury severity score

### The clinical outcome between the two groups

**Fig. 1:**
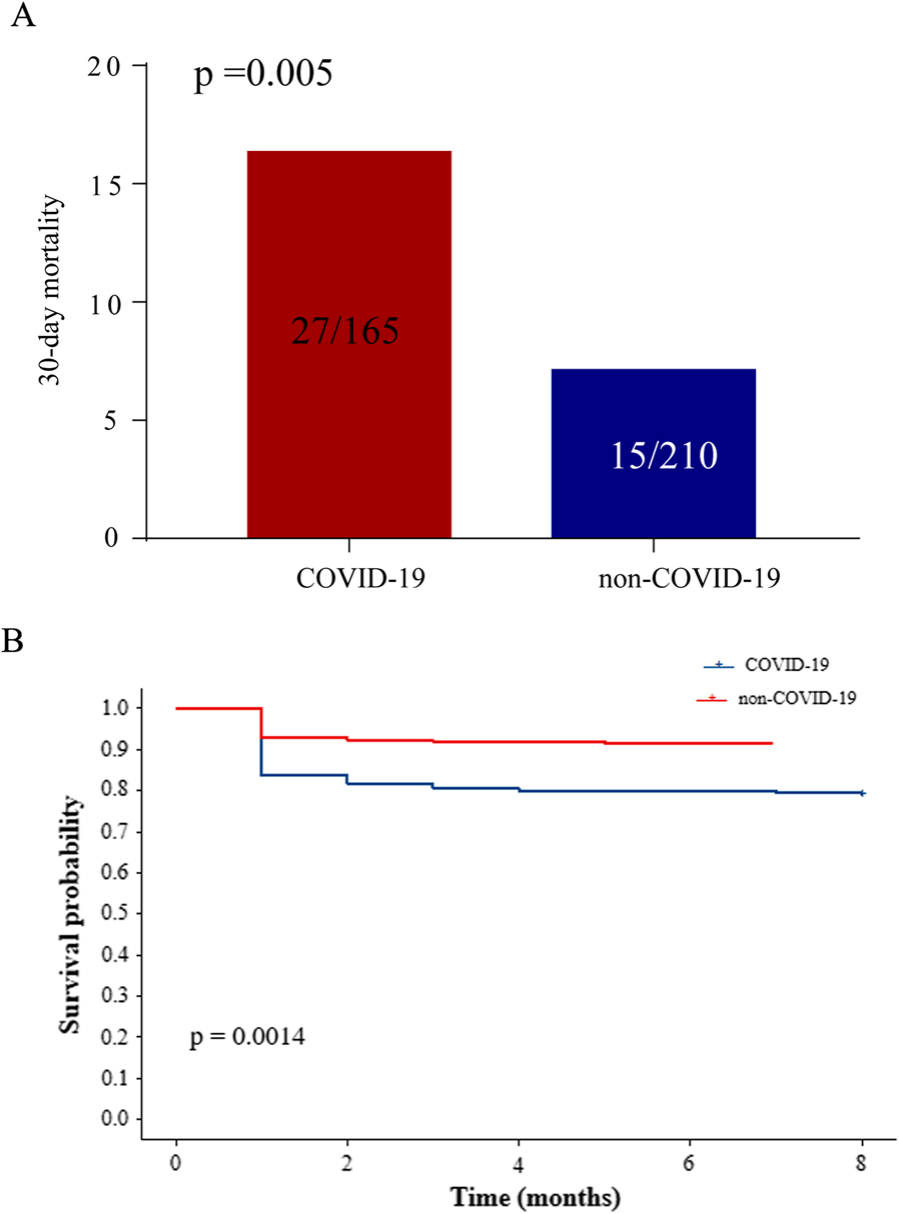
The clinical outcome between the two groups. **A** The 30-day mortality between the two groups. **B** Survival analysis

COVID-19 infection increases mortality in chest trauma. We compared the differences between the two groups. The 30-day mortality was 16.36% (27/165) in the chest trauma combined with COVID-19 infected group and 7.14% (15/210) in the chest trauma without COVID-19 infected group. COVID-19-infected patients showed a significantly higher 30-day mortality rate than noninfected patients (*p* = 0.005, Fig. 1A). Regarding clinical outcomes, chest trauma combined with COVID-19 was associated with a higher total 30-day mortality. Survival analysis also revealed that survival was better in the chest trauma without COVID-19-infected group than in the chest trauma combined with COVID-19-infected group (Fig. 1B).

### Hospital stays and hospitalization costs

**Fig. 2:**
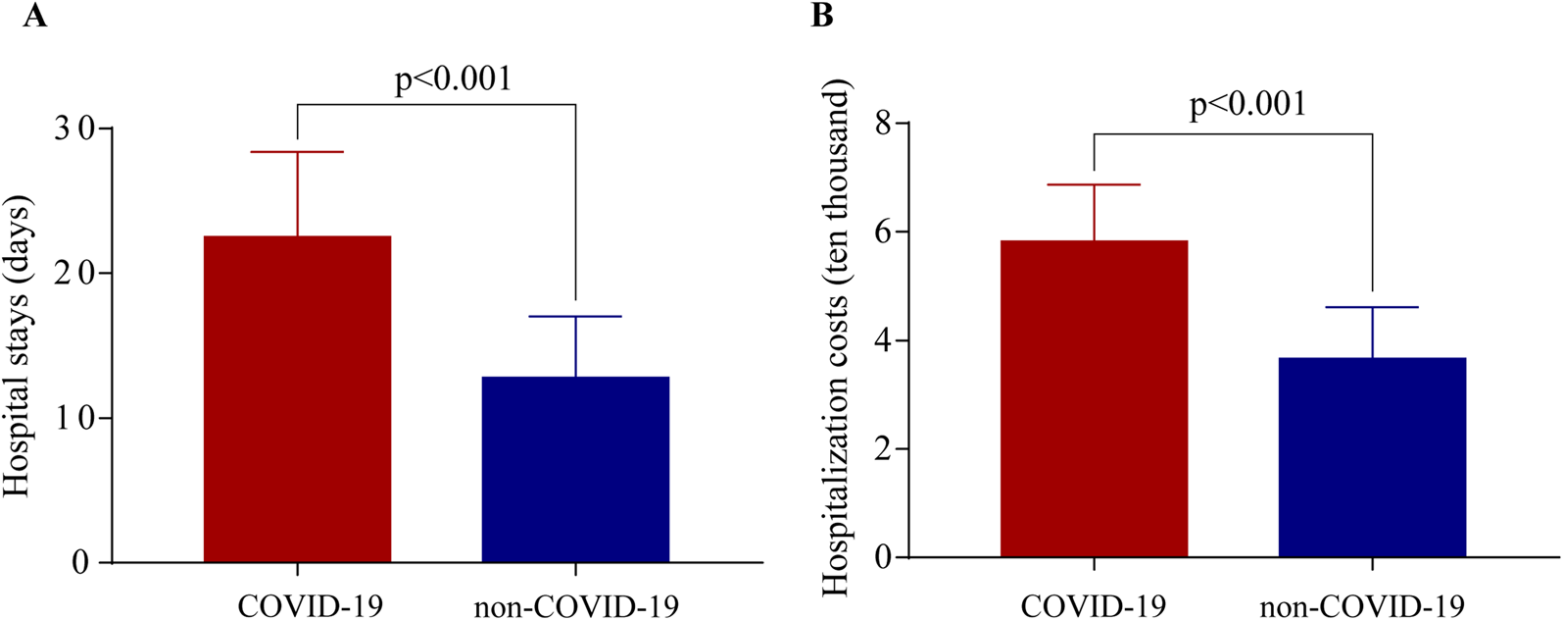
Hospital stays and hospitalization costs

In the chest trauma combined with COVID-19 infected patients, the average duration of stay was 22.5 ± 5.9 days, whereas the value for the non-COVID-19 infected patients was 12.8 ± 4.2 days, with a statistically significant difference (p < 0.001, Fig. 2a). The mean hospitalization expenditure of the COVID-19-infected patients was 58,200 RMB (Renminbi), which was much greater than the non-COVID-19 patient's cost of 36,700 RMB (*p* < 0.001, Fig. 2b). Additionally, both ICU length (8.46 ± 2.08 vs 7.11 ± 1.92, *p* < 0.001, Table 2) and mechanical ventilation rate (45.45% vs 30.48%, *p* = 0.003, Table 2) increased significantly in the COVID-19 infection group compared with the non-COVID-19 infection group. Hence, COVID-19 infection significantly increased chest trauma patients’ hospital stays and hospitalization costs.

**Table 2:** Comparison of complications between two groups

	COVID-19 (n = 165)	Non-COVID-19 (n = 210)	*p*
Pneumonia, no. (%)	128 (77.58%)	134 (63.80%)	0.004
Pulmonary embolism, no. (%)	17 (10.30%)	10 (4.76%)	0.039
DVT, no. (%)	55 (33.33%)	39 (18.57%)	0.001
7 days delirium, no. (%)	115 (69.70%)	97 (46.19%)	0.000
Respiratory failure, no. (%)	63 (38.18%)	52 (24.77%)	0.005
Heart failure, no. (%)	51 (30.91%)	37 (17.62%)	0.003
MODS, no. (%)	44 (26.67%)	28 (13.33%)	0.001
Neurological deficit, no. (%)	18 (10.91%)	25 (11.90%)	0.932
Abnormal liver enzymes, no. (%)	81 (49.10%)	93 (44.29%)	0.354
Diarrhea, no. (%)	64 (38.79%)	69 (32.86%)	0.233
Vomiting, no. (%)	59 (35.76%)	64 (30.48%)	0.280
ICU length (day), mean ± SD	8.46 ± 2.08	7.11 ± 1.92	0.001
Mechanical ventilation, no. (%)	75 (45.45%)	64 (30.48%)	0.003

### Complications between the two groups

During treatment for chest trauma, we also found more complications in patients with COVID-19. The incidence of pneumonia was elevated significantly in the COVID-19-infected patients compared with the non-COVID-19-infected patients (77.58% vs. 63.80, *p* = 0.004, Table 2). Pulmonary embolism (10.30% vs. 4.76%, *p* = 0.039) and deep vein thrombosis (DVT, 33.33% vs. 18.57%, *p* = 0.001) also increased in the COVID-19-infected patients, and there was a statistically significant difference between the two groups (*p* < 0.05, Table 2). Additionally, we also found that the incidences of 7-day delirium (69.70% vs. 46.19%, *p* < 0.001), respiratory failure (38.18% vs. 24.77%, *p* = 0.005), heart failure (30.91% vs. 17.62%, *p* = 0.003), and multiple organ dysfunction syndrome (MODS, 26.67% vs. 13.33%, *p* = 0.001) were increased in COVID-19-infected patients compared with non-COVID-19-infected patients. There were no significant differences between the two groups in abnormal liver enzymes (49.10% vs. 44.29%, *p* = 0.354), diarrhea (38.79 vs. 32.86, *p* = 0.233), vomiting (35.76% vs. 30.48%, *p* = 0.280), or neurological deficits (10.91% vs. 11.90%, *p* = 0.932). The results can be seen in Table 2.

## Discussion

This multicenter study examined the impact of the SARS-CoV-2 pandemic on common risk factors and treatment management for patients suffering from chest trauma. According to the present findings, we found no significant difference in the baseline patient characteristics between the two groups. Chest trauma combined with COVID-19 was associated with a higher total 30-day mortality. Chest trauma associated with COVID-19 also aggravated hospital stays and hospitalization costs. Additionally, COVID-19 combined with chest trauma increases complications during hospitalization, such as pneumonia, pulmonary embolism, 7-day delirium, respiratory failure, heart failure, and MODS.

Globally, the COVID-19 pandemic has impacted healthcare systems and practitioners. In December, COVID-19 spread in China after ending the zero-COVID policy. In the first month, almost all hospitalized patients were infected with COVID-19 before hospitalization or after admission. Overall, we did not find any differences between chest trauma combined with COVID-19 infection or not on baseline characteristics, such as age, sex, cause of disease, past medical history, and flail chest. We found that chest trauma combined with COVID-19 may result in a worse clinical outcome, more complications, and significantly increased chest trauma patients’ hospital stays and hospitalization costs. Patients with chest trauma often have lung infections. However, infection with COVID-19 can worsen lung infections, greatly increasing the rate of respiratory failure, and leading to longer hospital stays and larger hospitalization costs. Additionally, COVID-19 is often accompanied by fever and drug factors combined with gastrointestinal symptoms (diarrhea, poor appetite, etc.), resulting in increased water loss, and blood concentration is one of the risk factors for VTE. The use of a large number of hormones and immunoglobulins also causes blood stasis and aggravates the formation of VTE. A previous study reported that the levels of hypercoagulability indices, such as D-dimer, fibrinogen, and factor VIII, increased in all COVID-19 patients [9]. Recent research findings also reported that microvascular thrombi, neutrophil-platelet aggregates, endothelial inflammation, acquired antiphospholipid antibodies, hypercoagulability related to elevated coagulation factor levels, and reduced levels of endogenous anticoagulant proteins were potential mechanisms by which VTE occurs [10–12]. Hence, early anticoagulant therapy was necessary. To our knowledge, this was the first study to explore the clinical characteristics and outcomes of chest trauma patients during the COVID-19 pandemic.

Tilliridou et al. [13] reported that the 30-day mortality rate was higher in COVID-19 patients with pulmonary embolism than in those without pulmonary embolism. Multiple traumas combined with COVID-19 infection and positive CT findings can increase the risk for pulmonary complications [14]. Driessen et al. [15] also reported that there was a higher mortality rate during the SARS-CoV-2 pandemic than before. In the present study, we also found that more patients who died from chest trauma were infected with COVID-19, and the leading cause of death was multiple organ failure due to severe lung infection.

This study also had some limitations. First, the sample size was small, and larger sample studies are needed to explore the impact of the SARS-CoV-2 pandemic on the common risk factors and treatment management for patients suffering from chest trauma. Second, since we conducted a cross-sectional and retrospective study, we cannot demonstrate causality based on the data retrieved, and future studies examining the impact of a pandemic on intentional chest trauma should adopt a prospective, longitudinal design to allow the identification of risk factors and examination of causal links between variables. Third, the present study addresses only the short-term adverse events and efficacy of COVID-19 infection in chest trauma patients, and a long-term result is needed. Another limitation is related to missing data in the medical record database, including detailed clinical characteristics of chest trauma, such as the severity of injury and operation. Therefore, further and larger populations of chest trauma patients with COVID-19 should be investigated.

## Conclusion

According to the presented findings, chest trauma combined with COVID-19-infected patients had a higher total 30-day mortality, aggravated hospital stays and hospitalization costs, and increased complications during hospitalization, such as pneumonia, pulmonary embolism, 7-day delirium, respiratory failure, heart failure, and MODS. Additionally, the effects of longer-term follow-up were unclear. Therefore, further and larger populations of chest trauma patients with COVID-19 should be investigated. The present study also guides the future treatment of chest trauma combined with COVID-19 infection or other infectious diseases.

## Data Availability

The datasets used and analyzed in this study are available upon reasonable request from the corresponding author.
